# A Letter on the Physiological Doctrines of Marshall Hall, M. D.

**Published:** 1853-05

**Authors:** B. M. Wible

**Affiliations:** Louisville, Ky.


May, 1853.

---

Art. VII.—*A Letter on the Physiological Doctrines of Marshall Hall, M. D.* By B. M. Wible, M. D., of Louisville, Ky.

During the recent visit of Dr. Marshall Hall to our city, I had the pleasure of meeting him with a number of our physicians, when he performed for us some of those experiments from which he has educed that splendid discovery in physiological science of the distinct spinal system, and made it subservient to important improvements in pathology, diagnosis, and therapeutics.

I propose to give to the readers of your Journal, an account of the experiments made, although similar ones have been published before, and some of the profession are familiar with them.

By request, Dr. Hall made the experiments which prove the spinal cord to be the centre of all excito-motor actions, and that these are independent of sensation and volition.

A small frog was placed upon the table, and with a pair of scissors the head severed from the body, and the latter in a minute or two was in a state of complete repose. Dr. Hall afterwards asked those present if they thought the frog could remain perfectly and permanently quiet, after so severe an injury, if it retained sensation and powers of voluntary motion. It was conceded that the animal must be deprived of these.

Early after decapitation, the foot was pinched with a pair of forceps, and the limb contracted slowly; the shock from the injury having temporarily paralyzed the nervous energy; but in five or six minutes when the forceps was again applied the limb contracted actively. It was asked why did the limb contract if the frog possessed neither sensation nor voluntary motion? It had been agreed that it possessed neither of these, and therefore, the motive power must depend upon something else.

The frog was then turned upon its back, the abdomen laid open, and the heart, lungs, and alimentary organs, and the ganglionic system of nerves removed. The foot was again pinched, and the leg contracted actively. The conclusion was obvious that the motive power did not depend upon the ganglionic system of nerves.

A circular incision was next made around the lower part of the thigh, barely cutting through the skin, which was then stripped off towards the foot like a stocking. In pinching the limb thus denuded of the skin, no motion whatever could be elicited. In removing the skin from the leg, a small portion of it was left attached to the foot, which being pinched caused the leg to contract, but after its removal no contractions could be produced by pinching the leg below the place where the circular incision had been made. These experiments prove the skin to be a point in the arc of the nervous course in these phenomena.

Next, the spinal nerves, given off to the limb, (which had not been deprived of the skin, and which still retained its contractile power) were divided close to their origin in the spinal cord, and instantly the limb became flaccid, and the pinching failed to induce the least contraction.

Subsequently the experiment was varied by destroying the spinal cord in a vigorous frog, and when the destruction was completed, (which was done in a few seconds) not the least movement could be induced by pinching any of the limbs; on the contrary they were as flaccid and passive as if the animal had been dead a month.

These experiments prove the spinal cord to be essential to those excited muscular movements which are independent of sensation and voluntary motive power.

Afterwards like experiments were made upon the snake, and the independence of excited muscular movement from sensation or pain was clearly shown. After the snake was decapitated it was hung up by means of a cord tied around the neck, and, at first, its own motions kept up excited impressions, probably by the action of the scales upon the skin; but soon afterwards it became perfectly still. But touching it again upon the tail, or any part of the body, it would be thrown into contortions, which subsiding, the same experiments could be again and again repeated, productive of the same results.

When the snake was taken down, and placed upon the table, muscular action was again excited. It was soon, though, in repose, and would remain so permanently, unless excited by external influences, and this, it was stated, had been fully demonstrated by experiments made upon animals placed under a bell glass where the air and other disturbing causes were excluded.

In man the same phenomenon is observable as in the frog or snake. In cases of paralysis of the lower extremities, where both sensation and powers of voluntary motion are lost, owing to disease, compression, or division of the spinal cord, active movements of the limbs may be produced by irritation applied to the soles of the feet, or by plucking the hairs on the leg.

The conclusion from these experiments and observations is, that the nerves of excited muscular movement have their *origin* in the skin, mucous membrane, &c., their *centre* in the spinal cord, and their *destination* in the muscles. An arc is thus formed, having one extremity in the skin, the other in the muscle, and the centre in the spinal cord.

The *passage* of the nerve from the skin to the spinal cord is denominated the *course;* and that from the spinal cord to the muscle, the *reflex course* in these phenomena.

The nerve passing from the skin to the spinal cord is called the *eisodic* nerve, and that from the spinal cord to the muscle, the *exodic* nerve.

It has been disputed that the spinal cord is the centre of excited muscular movement, particularly by Dr. Dowler, of New Orleans; but any one who will read his accounts of experiments made on the alligator, and then witness those made on the frog, or snake, by Dr. Marshall Hall, will be convinced that Dr. Dowler was not qualified to interpret what he observed.

The importance of these spinal functions, their bearing on diagnosis and therapeutics was discussed during the evenings spent with Dr. Marshall Hall, and may be the subject of another letter for some future number of your journal. Dr. Marshall Hall's aim in showing these experiments, and in the examination of a patient, is, according to an expression of his own, "to bring out the facts, and make *them* speak," and certainly the effect is at once highly interesting, impressive and convincing.

May, 1853.

